# Child Excess Weight Status, Adult Excess Weight Status, and Cardiometabolic Risk Profile

**DOI:** 10.3389/fped.2020.00301

**Published:** 2020-06-09

**Authors:** Hui Fan, Qi Zhu, Xingyu Zhang

**Affiliations:** ^1^Department of Preventive Medicine, North Sichuan Medical College, Nanchong, China; ^2^Applied Biostatistics Laboratory, University of Michigan School of Nursing, Ann Arbor, MI, United States

**Keywords:** weight status, cardiometabolic risk factors, childhood, adulthood, cohort

## Abstract

**Background:** The potential effects of excess weight status in childhood on later adult cardiometabolic risk factors have been undetermined in a Chinese population. Additionally, the potential mitigation of these effects if adult weight status returns to normalcy has been unresolved. Accordingly, we aimed to assess the association of childhood excess weight status and its long-term change with adult cardiometabolic risk factors.

**Methods:** A cohort study from the China Health and Nutrition Survey 1991–2009 consisted of 541 participants who were measured in childhood (≥6 and <18 years) and underwent laboratory assessment in adulthood (≥18 years). In childhood, the participants were classified into four groups as age-sex-specific body mass index (BMI) z-score quartiles. The adult cardiometabolic risk factors included overweight and obesity, hypertension, high total cholesterol, high triglyceride, low high-density lipoprotein cholesterol, high low-density lipoprotein cholesterol, and high hemoglobin A1c.

**Results:** The prevalence was 61.0, 36.2, and 19.0% for ≥1, 2, and 3 cardiometabolic risk factors, respectively, with a mean 14.9-year follow-up. There was a significant trend in the progression of the number of adult cardiometabolic risk factors across childhood BMI quartiles (*P* < 0.001). Additionally, participants with childhood BMI z-scores ≥ 75th percentile and adult BMI z-scores < 75th percentile did not have increased cardiometabolic risks compared with those with both childhood and adulthood BMI z-scores < 75th percentile.

**Conclusions:** Our findings revealed that child excess weight status increased adult cardiometabolic risks. However, the effects of excess weight status in childhood on adult cardiometabolic risk factors were mitigated if adult weight status returned to normalcy.

## Introduction

Cardiovascular disease (CVD) remains the leading cause of mortality across the globe (1). Cardiometabolic risk factors increase the risk of CVD, which results in a future burden of CVD (2). Controlling the epidemic of cardiometabolic risk factors has proven to be one effective strategy for the prevention of CVD.

Cardiometabolic risk factors tend to cluster and include excess weight status, high blood pressure, dyslipidemia, and hyperglycemia (3–5). Excess weight status causes inflammation and insulin resistance, which play a key role in the onset and clustering of cardiometabolic risk factors (6). Excess weight status is the easiest cardiometabolic risk factor to identify, and often is attained in early life (3–6).

The worldwide prevalence of childhood excess weight status has increased at an alarming rate in recent years (7). Observational studies have shown that children with excess weight status have an increased risk of cardiometabolic risk factors in adulthood (8, 9). Furthermore, evidence regarding the impact of the change in weight status from childhood to adulthood on health consequences has revealed that the adverse health consequences can be reversed if children with excess weight status attain normal weight as adults (10–13). However, few similar studies have been conducted in China. As industrialization and urbanization have accelerated and the population has aged, CVD has become a major public health challenge in China. Reliable information is essential for the development of national health policies for the prevention and control of CVD (14). Consequently, we aimed to assess the association of childhood weight status and its long-term change with cardiometabolic risk factors in early adulthood based on the China Health and Nutrition Survey (CHNS).

## Materials and Methods

### Study Population

Launched in 1989, the CHNS is an ongoing, open, and population-based longitudinal cohort study that is designed to examine health and nutrition status in the Chinese population (15). The survey sample was drawn with the use of a stratified multistage cluster method. As part of the CHNS, a follow-up survey is conducted every 2–4 years. Participants are asked to complete an interview questionnaire and a physical examination at each survey instance (15). Notably, fasting blood was collected for the first time in the 2009 survey (16, 17). The survey procedures used for the CHNS are described in detail elsewhere (15). This study was approved by the Institutional Review Board at the University of North Carolina at Chapel Hill, Institute of Nutrition and Food Safety, China Centers for Disease Control, and the China-Japan Friendship Hospital, Ministry of Health and China. All participants or their guardians provided written informed consent.

We established a cohort study from childhood (≥6 and <18 years) to adulthood (≥18 years) based on the CHNS. To ensure adequate follow-up length, individuals with a full record of key information (sex, age, blood pressure, weight, and height) on the first measurement during childhood, collected before the 2009 survey, were included in the present study. The follow-up survey was conducted among adult participants in the 2009 survey. In total, 660 participants were eligible. However, 119 participants did not undergo laboratory assessment in 2009. As a result, the cohort consisted of 541 individuals with a full record of key information during childhood before 2009, and had a full record of key information and had undergone laboratory assessment during adulthood in 2009.

### General Examinations and Laboratory Assessment

Sex, age, and adult risk factors (smoking and alcohol consumption) were collected by self-administered questionnaire. Weight and height were measured by trained workers, respectively. Body mass index (BMI) was calculated as weight in kilograms divided by the square of height in meters. Blood pressure (BP) was measured using certified mercury sphygmomanometers. Three consecutive BP measurements were obtained, and the average of the latter two measurements was used for further analyses.

Fasting blood was collected in the 2009 survey. The methods of blood sample collection and preservation, the measurement procedures, the measurement equipment, and the test method are described in detail in several previous publications (16, 17). Total cholesterol (TC), triglyceride (TG), high-density lipoprotein cholesterol (HDL-C), low-density lipoprotein cholesterol (LDL-C), and hemoglobin A1c (HbA1c) were measured.

### Definitions

BMI was used to evaluate childhood weight status (18). Childhood overweight and obesity were defined as BMI ≥ corresponding sex- and age-specific overweight cutoffs presented in the national reference for Chinese children (19). Adulthood overweight and obesity were defined as BMI ≥ 24 kg/m^2^ (20). Childhood elevated BP was defined as BP ≥ 90th percentile for sex, age, and height or 120/80 mm Hg as per the BP reference for Chinese children (21, 22). Adulthood hypertension was defined as BP ≥ 140/90 mm Hg or taking anti-hypertension medications according to the 2018 Chinese Guidelines for Prevention and Treatment of Hypertension (21). Adulthood high TC was defined as TC ≥ 200 mg/dL, high TG as ≥150 mg/dL, high LDL-C as ≥130 mg/dL, and low HDL-C as <40 mg/dL in terms of the 2016 Chinese Guidelines for the Prevention and Treatment of Dyslipidemia in adults (23). High HbA1c was defined as ≥5.6% (16). The number of individual cardiovascular risk factors (overweight and obesity, hypertension, high TC, high TG, high LDL-C, low HDL-C, and high HbA1c) in adulthood was calculated to assess the cardiovascular risk profile.

### Statistical Analysis

After adjusting for sex and age by regression residual analyses, childhood BMI was standardized using the Z-transformation (mean = 0, SD = 1). The participants were categorized into four groups as quartiles of childhood BMI z-scores. The data between groups were presented as means (SDs), medians (interquartile ranges), or frequencies (%) as appropriate. The differences between groups were tested using either analysis of variance, the chi-square test, Fish's exact probabilities, or the nonparametric test. Poisson models with robust standard errors were used to calculate relative risks (RRs) and 95% confidence intervals (CIs) with adjustment for covariates, and to investigate the relation of childhood weight status to cardiovascular risk factors in adulthood (24). The trend across the quartiles was tested using quartiles as a continuous ordinal variable.

Individuals were classified into 4 groups based on combinations of childhood and adulthood weight status: childhood BMI z-scores < 75th percentile and adult BMI z-scores < 75th percentile (Group 1), childhood BMI z-scores < 75th percentile and adult BMI z-scores ≥ 75th percentile (Group 2), childhood BMI z-scores ≥ 75th percentile and adult BMI z-scores < 75th percentile (Group 3), childhood BMI z-scores ≥ 75th percentile and adult BMI z-scores ≥ 75th percentile (Group 4). Differences in the number of cardiovascular risk factors between groups were tested using the chi-square test. Covariate-adjusted Poisson models were used to assess the association between weight status change from childhood to adulthood and the number of cardiovascular risk factors in adulthood.

We used SAS 9.4 (SAS Institute Inc., Cary, NC, USA) to conduct the analyses and considered a two-tailed *P* < 0.05 to be statistically significant.

## Results

The present study included 541 participants (males, 71.9%) from the CHNS 1991–2009. The participants' age ranged from 6 to 17 years (mean age, 11.4 years) in childhood and from 18 to 35 years (mean age, 26.2 years) in adulthood. The mean follow-up duration was 14.9 years (median, 16.0 years; range, 3–19 years).

Table 1 summarizes the characteristics of all participants. The prevalence of overweight and obesity in childhood and adulthood was 9.1 and 24.0%, respectively. Adult hypertension, high TC, high TG, high LDL-C, low HDL-C, and high HbA1c prevalences were 7.8, 16.8, 23.7, 15.7, 14.8, and 25.7%, respectively. 61.0, 36.2, and 19.0% of all individuals had ≥1, ≥2, and ≥3 cardiovascular risk factors in adulthood, respectively. Additionally, compared with individuals in the first quartile of childhood BMI, those in the fourth quartile tended to have more cardiovascular risk factors in adulthood.

**Table 1:** Characteristics of the participants by childhood BMI quartiles.

	**All participants (*n* = 541)**	**Childhood BMI quartiles**				
		**Q1 (*n* = 135)**	**Q2 (*n* = 136)**	**Q3 (*n* = 135)**	**Q4 (*n* = 135)**	***P***
Male (%)	71.9	71.9	72.1	71.9	71.9	0.999
**Childhood**						
Age (years)	11.4 (3.2)	12.0 (2.9)	10.8 (2.9)	11.1 (3.6)	11.7 (3.4)	0.011
BMI (kg/m^2^)	17.1 (2.7)	15.0 (1.5)	16.0 (1.4)	17.2 (1.7)	20.3 (2.7)	<0.001
Overweight and obesity (%)	9.1	0.0	0.0	0.0	36.3	<0.001
SBP (mm Hg)	95.6 (13.2)	94.6 (12.2)	93.8 (11.7)	94.6 (12.6)	99.4 (15.3)	0.001
DBP (mm Hg)	62.4 (10.1)	61.1 (9.4)	60.4 (9.2)	62.6 (9.4)	65.3 (11.6)	<0.001
Elevated BP (%)	17.9	13.3	14.7	17.0	26.7	0.019
**Adulthood**						
Age (years)	26.2 (5.0)	26.7 (4.9)	26.0 (4.6)	26.1 (5.0)	26.3 (5.4)	0.696
BMI (kg/m^2^)	21.9 (3.4)	19.9 (2.6)	21.6 (3.0)	22.5 (2.9)	23.7 (3.6)	<0.001
Overweight and obesity (%)	24.0	5.9	15.4	30.4	44.4	<0.001
SBP (mm Hg)	113.5 (11.7)	110.0 (11.1)	113.0 (11.1)	114.6 (12.2)	116.3 (11.4)	<0.001
DBP (mm Hg)	75.1 (9.3)	71.8 (8.4)	74.6 (9.0)	75.9 (8.8)	77.9 (9.9)	<0.001
Hypertension (%)	7.8	4.4	6.6	9.6	10.4	0.234
Smoking (%)						0.269
Never	60.3	60.7	59.6	54.1	66.7	
Former	2.2	1.5	1.5	2.2	3.7	
Current	37.5	37.8	39.0	43.7	29.6	
Drinking (%)						0.814
Never	52.7	48.9	52.9	53.3	55.6	
Almost every day	4.1	3.0	5.9	5.2	2.2	
3–4 times/week	4.8	5.2	7.4	3.7	3.0	
1–2 times/week	11.8	11.1	9.6	12.6	14.1	
1–2 times/month	15.3	17.8	14.0	14.8	14.8	
<1 time/month	11.3	14.1	10.3	10.4	10.4	
TC (mg/dl)	170.2 (36.4)	167.6 (34.1)	173.1 (38.2)	171.2 (37.6)	168.8 (35.8)	0.600
High TC (%)	16.8	14.8	18.4	14.8	19.3	0.662
TG (mg/dl)	99.2 (66.4–147.9)	92.1 (66.4–127.6)	99.2 (63.8–139.3)	103.6 (68.2–172.7)	106.3 (67.3–155.9)	0.180
High TG (%)	23.7	17.8	18.4	32.6	25.9	0.012
LDL-C (mg/dl)	101.3 (33.4)	96.6 (29.6)	103.9 (37.0)	103.0 (32.8)	101.5 (33.7)	0.279
High LDL-C (%)	15.7	11.9	17.6	14.8	18.5	0.425
HDL-C (mg/dl)	53.0 (13.2)	55.1 (12.0)	55.0 (14.4)	51.8 (13.7)	50.2 (12.1)	0.003
Low HDL-C (%)	14.8	9.6	13.2	18.5	17.8	0.135
HbA1c (%)	5.3 (0.6)	5.2 (0.6)	5.2 (0.4)	5.3 (0.4)	5.4 (0.7)	0.008
High HbA1c (%)	25.7	20.0	22.8	28.9	31.1	0.128
≥1 cardiovascular risk factors (%)	61.0	44.4	56.6	68.9	74.1	<0.001
≥2 cardiovascular risk factors (%)	36.2	22.2	31.6	43.7	47.4	<0.001
≥3 cardiovascular risk factors (%)	19.0	10.4	16.2	23.7	25.9	0.004

*BMI, body mass index; BP, blood pressure; DBP, diastolic blood pressure; HDL-C, high-density lipoprotein cholesterol; LDL-C, low-density lipoprotein cholesterol; SBP, systolic blood pressure; TC, total cholesterol; TG, triglyceride; HbA1c, hemoglobin A1c*.

*Data are presented as means (SDs), medians (interquartile ranges), or frequencies (%) as appropriate*.

*Differences between continuous variables were compared using the analysis of variance or nonparametric test*.

*Differences between categorical variables were compared using the χ^2^ test or Fish's exact probabilities*.

Table 2 presents the association between childhood BMI and adult cardiometabolic risk profile. Participants in the fourth quartile of childhood BMI had an increased risk of cardiovascular risk factors in comparison with those in the first quartile after adjusting for sex, childhood age, and elevated BP (Model 1). RR did not vary significantly after adjusting further for the follow-up duration, adult smoking, and drinking (Model 2). There was a significant trend in the progression of the number of adult cardiometabolic risk factors across the quartiles of childhood BMI in the fully adjusted model (*P* for trend <0.001).

**Table 2:** Association of childhood BMI with adult cardiometabolic risk profile.

	**Model 1: RR (95%CI)**	**Model 2: RR (95%CI)**
**Outcome:** **≥1 cardiovascular risk factors**		
Continuous childhood BMI	1.15 (1.08–1.23)^***^	1.16 (1.09–1.24)^***^
**Childhood BMI quartiles**		
First quartile	Ref	Ref
Second quartile	1.30 (1.03–1.64)^*^	1.27 (1.01–1.59)^*^
Third quartile	1.56 (1.26–1.94)^***^	1.54 (1.24–1.90)^***^
Fourth quartile	1.64 (1.33–2.02)^***^	1.64 (1.33–2.01)^***^
*P* for trend	<0.001	<0.001
**Outcome:** **≥2 cardiovascular risk factors**		
Continuous childhood BMI	1.25 (1.13–1.38)^***^	1.28 (1.16–1.42)^***^
**Childhood BMI quartiles**		
First quartile	Ref	Ref
Second quartile	1.43 (0.96–2.12)	1.37 (0.93–2.02)
Third quartile	1.97 (1.38–2.82)^***^	1.94 (1.35–2.77)^***^
Fourth quartile	2.11 (1.47–3.02)^***^	2.11 (1.47–3.01)^***^
*P* for trend	<0.001	<0.001
**Outcome:** **≥3 cardiovascular risk factors**		
Continuous childhood BMI	1.32 (1.15–1.51)^***^	1.38 (1.19–1.61)^***^
**Childhood BMI quartiles**		
First quartile	Ref	Ref
Second quartile	1.60 (0.86–2.97)	1.52 (0.82–2.84)
Third quartile	2.30 (1.31–4.04)^**^	2.24 (1.27–3.98)^**^
Fourth quartile	2.34 (1.33–4.13)^**^	2.38 (1.34–4.24)^**^
*P* for trend	<0.001	<0.001

*BMI, body mass index; CI, confidence interval; RR, relative risk*.

*Continuous childhood BMI was transformed into age- and sex-specific Z-scores*.

*Model 1: adjusted for sex, childhood age and elevated blood pressure; Model 2: additionally adjusted for the length of follow-up and adult risk factors (smoking and drinking)*.

* *P <0.05*;

** *P <0.01*;

*** *P <0.001*.

**Figure 1:**
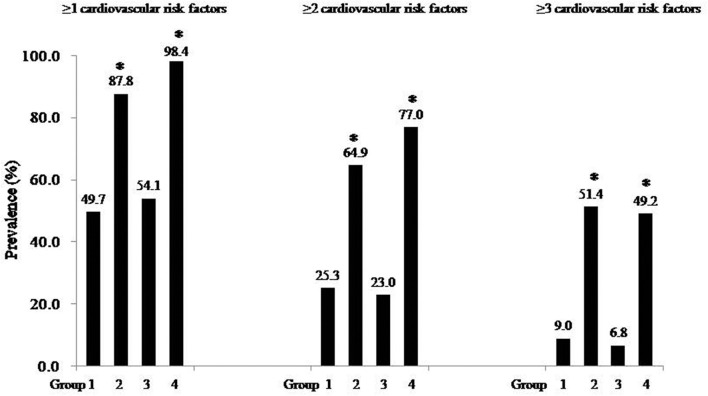
Prevalence of cardiometabolic risk factors among four groups. Group 1: childhood BMI z-scores < 75th percentile and adult BMI z-scores < 75th percentile; Group 2: childhood BMI z-scores < 75th percentile and adult BMI z-scores ≥ 75th percentile; Group 3: childhood BMI z-scores ≥ 75th percentile and adult BMI z-scores < 75th percentile; Group 4: childhood BMI z-scores ≥ 75th percentile and adult BMI z-scores ≥ 75th percentile. *P < 0.001 (Compared with group 1).

Figure 1 describes the prevalence of cardiometabolic risk factors among the four groups as defined in the combinations of childhood and adulthood weight status. Interestingly, the difference in the prevalence of cardiometabolic risk factors between the participants with both child and adult BMI z-scores < 75th percentile and those with child BMI z-scores ≥ 75th percentile and adult BMI z-scores < 75th percentile was not significant (*P* > 0.05).

Table 3 shows the impact of weight status change from childhood to early adulthood on cardiometabolic risk factors. In comparison with the participants who had both child and adult BMI z-scores < 75th percentile, those with child BMI z-scores ≥ 75th percentile and adult BMI z-scores < 75th percentile had no increased risk of adult cardiometabolic risk factors (all *P*_s_ > 0.05).

**Table 3:** Weight status change from childhood to adulthood and adult cardiometabolic risk factors.

	**Outcome:** **≥1 cardiovascular risk factors**		**Outcome:** **≥2 cardiovascular risk factors**		**Outcome:** **≥3 cardiovascular risk factors**	
	**RR (95%CI)^†^**	***P***	**RR (95%CI)^†^**	***P***	**RR (95%CI)^†^**	***P***
Group 1 (*n* = 332)	Ref		Ref		Ref	
Group 2 (*n* = 74)	1.80 (1.58–2.06)	<0.001	2.57 (2.03–3.25)	<0.001	6.17 (4.12–9.23)	<0.001
Group 3 (*n* = 74)	1.10 (0.87–1.38)	0.440	0.94 (0.60–1.48)	0.801	0.77 (0.32–1.86)	0.567
Group 4 (*n* = 61)	1.94 (1.71–2.19)	<0.001	3.00 (2.36–3.80)	<0.001	5.31 (3.48–8.08)	<0.001

*CI, confidence interval; RR, relative risk*.

† *Adjusted for sex, childhood age, and elevated blood pressure, follow-up duration, adult smoking, and drinking*.

*Group 1: childhood BMI z-scores < 75th percentile and adult BMI z-scores < 75th percentile; Group 2: childhood BMI z-scores < 75th percentile and adult BMI z-scores ≥ 75th percentile; Group 3: childhood BMI z-scores ≥ 75th percentile and adult BMI z-scores < 75th percentile; Group 4: childhood BMI z-scores ≥ 75th percentile and adult BMI z-scores ≥ 75th percentile*.

We excluded 134 participants with child elevated BP or overweight (including obesity) to perform a sensitivity analysis. The sensitivity analysis results were similar (Tables S1, S2). In addition, we used the national reference for Chinese children and Asia adult reference (BMI ≥ 24 kg/m^2^) to define child and adult overweight (including obesity), respectively (19, 20). We performed a sensitivity analysis and obtained similar results (Table S3).

## Discussion

We revealed that childhood excess weight status increased adult cardiometabolic risks based on a cohort study with a mean 14.9-year follow-up from the CHNS 1991–2009. Moreover, our results suggested that the effects of excess weight status in childhood on adult cardiometabolic risk factors were mitigated if adult weight status returned to normalcy. Our findings have important implications for the prevention and control of CVD in the Chinese population.

Child excess weight status increased the risk of early-onset and clustering of future cardiometabolic risk factors (10). Previous studies reported that the number of cardiometabolic risk factors increased with the extent of childhood excess weight status (8). Our results were consistent with the previous findings, indicating that childhood excess weight status was an important determinant in the development of cardiometabolic risk factors. In addition, several prospective cohort studies began in childhood presented BMI trajectory and incremental area under the growth curve to obtain similar findings (25, 26). Moreover, childhood excess weight status predicted significantly future CVD and both cardiovascular and all-cause mortality across the life span (9, 27–29).

Maintaining an ideal weight in early life is an effective means for the prevention and control of future CVD. The International Childhood Cardiovascular Cohort (i3C) Consortium pooled data from four prospective cohort studies in the Western population, and revealed that children with excess weight status who achieved an ideal weight in adulthood did not have an increased risk of cardiovascular risk factors (10). Moreover, ample evidence demonstrated that the effect of childhood adiposity on cardiometabolic profile was mediated by current BMI (11–13). Consistent with previous publications, the present study showed that the long-term adverse effects of childhood excess weight status can be reversed if weight status returned to normalcy in adulthood in the Chinese population. Our findings were also supported by existing evidence from the Chinese population, which showed that a decrease in excess weight status from childhood to adulthood was related to significant reductions in the risks of hypertension, metabolic syndrome, nonalcoholic fatty liver disease, subclinical atherosclerosis, arterial stiffness, and left ventricular hypertrophy (30–33).

There were several limitations in our study. First, data regarding blood glucose and lipid levels in childhood were not collected, which may affect our results. However, the sensitivity analysis results did not vary significantly after excluding 134 participants with childhood elevated BP or overweight (including obesity). In addition, pediatric overweight and obesity were equivalent to metabolic syndrome in terms of the power to predict adult adverse health consequence (9). Second, this observational study was not able to assess the causality between weight status change from childhood to adulthood and cardiometabolic risk factors. Third, 18% (119/660) of participants were lost to follow-up. However, no significant difference in childhood characteristics, except systolic BP, was observed between those who were included in the present study and those who were lost to follow-up (Table S4). Fourth, the sample size was insufficient to perform stratification analysis. Fifth, the number of female participants was small, which may affect our findings.

In conclusion, the current study demonstrated that childhood excess weight status is positively associated with adult cardiometabolic risk factors. Given that China is facing an excess weight epidemic in childhood (34), our results underscore the importance for weight management in early life in regard to the prevention and control of adult cardiometabolic risk factors and CVD.

## Data Availability Statement

The raw data supporting the conclusions of this article will be made available by the authors, without undue reservation, to any qualified researcher.

## Ethics Statement

The studies involving human participants were reviewed and approved by the Institutional Review Board at the University of North Carolina at Chapel Hill, Institute of Nutrition and Food Safety, China Centers for Disease Control and the China-Japan Friendship Hospital, Ministry of Health and China. Written informed consent to participate in this study was provided by the participants' legal guardian/next of kin.

## Author Contributions

HF conceptualized and designed the study, carried out the initial analyses, drafted the initial manuscript, and reviewed and revised the manuscript. QZ and XZ critically reviewed and revised the manuscript. All authors approved the final manuscript for submission.

## Conflict of Interest

The authors declare that the research was conducted in the absence of any commercial or financial relationships that could be construed as a potential conflict of interest.

## Abbreviations

BMI: body mass index
BP: blood pressure
CVD: cardiovascular disease
DBP: diastolic blood pressure
HbA1c: hemoglobin A1c
HDL-C: high-density lipoprotein cholesterol
LDL-C: low-density lipoprotein cholesterol
SBP: systolic blood pressure
TC: total cholesterol
TG: triglycerides.
