# Supplementary material for: Child Excess Weight Status, Adult Excess Weight Status, and Cardiometabolic Risk Profile
Source: Front Pediatr. 2020 Jun 9;8:301. doi: 10.3389/fped.2020.00301 (PMC7295978; doi:10.3389/fped.2020.00301)
Supplement: Supplementary file 1 [file Data_Sheet_1.docx]

| Table S1. Association between childhood BMI and adult cardiometabolic risk profile | |
| --- | --- |
|  | RR (95%CI) † |
| Outcome: ≥1 cardiovascular risk factors |  |
| Continuous childhood BMI | 1.17 (1.08-1.27)*** |
| Childhood BMI quartiles |  |
| First quartile (n=101) | Ref |
| Second quartile (n=103) | 1.18 (0.88-1.56) |
| Third quartile (n=102) | 1.54 (1.19-1.99)** |
| Fourth quartile (n=101) | 1.59 (1.24-2.05)*** |
| *P* for trend | <0.001 |
| Outcome: ≥2 cardiovascular risk factors |  |
| Continuous childhood BMI | 1.28 (1.13-1.45)*** |
| Childhood BMI quartiles |  |
| First quartile (n=101) | Ref |
| Second quartile (n=103) | 1.14 (0.72-1.81) |
| Third quartile (n=102) | 1.91 (1.26-2.89)** |
| Fourth quartile (n=101) | 1.76 (1.15-2.69)** |
| *P* for trend | <0.001 |
| Outcome: ≥3 cardiovascular risk factors |  |
| Continuous childhood BMI | 1.30 (1.06-1.60)* |
| Childhood BMI quartiles |  |
| First quartile (n=101) | Ref |
| Second quartile (n=103) | 1.11 (0.51-2.45) |
| Third quartile (n=102) | 2.31 (1.17-4.54)* |
| Fourth quartile (n=101) | 1.97 (0.96-4.05) |
| *P* for trend | 0.010 |
| BMI, body mass index; CI, confidence interval; RR, relative risk.  Continuous childhood BMI was transformed into age- and sex-specific Z-scores.  †Adjusted for sex, childhood age, the length of follow-up and adult risk factors (smoking and drinking).  **P<*0.05; ***P*<0.01; ****P*<0.001 | |
|  |  |
|  |  |

| Table S2. Weight status change from childhood to adulthood and adult cardiometabolic risks | | | | | | | | |
| --- | --- | --- | --- | --- | --- | --- | --- | --- |
|  | Outcome: ≥1 cardiovascular risk factors | |  | Outcome: ≥2 cardiovascular risk factors | |  | Outcome: ≥3 cardiovascular risk factors | |
|  | RR (95%CI)† | *P* |  | RR (95%CI)† | *P* |  | RR (95%CI)† | *P* |
| Group 1 (n=247) | Ref |  |  | Ref |  |  | Ref |  |
| Group 2 (n=59) | 1.98 (1.67-2.34) | <0.001 |  | 2.59 (1.93-3.48) | <0.001 |  | 7.73 (4.66-12.84) | <0.001 |
| Group 3 (n=59) | 1.13 (0.86-1.49) | 0.374 |  | 0.85 (0.50-1.44) | 0.544 |  | 0.73 (0.23-2.32) | 0.594 |
| Group 4 (n=42) | 2.03 (1.72-2.39) | <0.001 |  | 2.75 (2.06-3.68) | <0.001 |  | 5.65 (3.14-10.14) | <0.001 |
| CI, confidence interval; RR, relative risk.  †Adjusted for sex, childhood age, the length of follow-up and adult risk factors (smoking and drinking).  Group 1: childhood BMI z-scores<75th percentile and adult BMI z-scores<75th percentile; Group 2: childhood BMI z-scores<75th percentile and adult BMI z-scores≥75th percentile; Group 3: childhood BMI z-scores≥75th percentile and adult BMI z-scores<75th percentile; Group 4: childhood BMI z-scores≥75th percentile and adult BMI z-scores≥75th percentile. | | | | | | | | |
|  |  |  |  |  |  |  |  |  |
|  |  |  |  |  |  |  |  |  |

| Table S3. Weight status change from childhood to adulthood and adult cardiometabolic risks* | | | | | | | | |
| --- | --- | --- | --- | --- | --- | --- | --- | --- |
|  | Outcome: ≥1 cardiovascular risk factors | |  | Outcome: ≥2 cardiovascular risk factors | |  | Outcome: ≥3 cardiovascular risk factors | |
|  | RR (95%CI)† | *P* |  | RR (95%CI)† | *P* |  | RR (95%CI)† | *P* |
| Group 1 (n=388) | Ref |  |  | Ref |  |  | Ref |  |
| Group 2 (n=104) | 1.97 (1.77-2.21) | <0.001 |  | 3.09 (2.48-3.84) | <0.001 |  | 8.16 (5.41-12.31) | <0.001 |
| Group 3 (n=23) | 1.36 (0.97-1.90) | 0.078 |  | 1.13 (0.51-2.53) | 0.764 |  | 0.89 (0.12-6.39) | 0.904 |
| Group 4 (n=26) | 2.06 (1.81-2.34) | <0.001 |  | 4.02 (3.10-5.22) | <0.001 |  | 10.51 (6.61-16.73) | <0.001 |
| CI, confidence interval; RR, relative risk.  †Adjusted for sex, childhood age and elevated blood pressure, the length of follow-up and adult risk factors (smoking and drinking).  * Child overweight and obesity was defined as the national reference for Chinese children; adult overweight and obesity was defined as BMI≥24 kg/m^2^.  Group 1: childhood and adult normal weight; Group 2: childhood normal weight and adult overweight (including obesity); Group 3: childhood overweight (including obesity) and adult normal weight; Group 4: childhood and adult overweight (including obesity). | | | | | | | | |
|  |  |  |  |  |  |  |  |  |
|  |  |  |  |  |  |  |  |  |

| Table S4. Comparison of childhood characteristics between participants who were followed up successfully and those who were lost to follow-up | | | |
| --- | --- | --- | --- |
|  | Participants (n=541) | [Loss](javascript:;) [to](javascript:;) [follow-up](javascript:;)(n=119) | *P*† |
| Males (%) | 71.9 | 79.8 | 0.077 |
| Age (years) | 11.4 (3.2) | 11.4 (3.3) | 0.877 |
| BMI (kg/m^2^) | 17.1 (2.7) | 17.6 (3.1) | 0.114 |
| SBP (mm Hg) | 95.6 (13.2) | 98.4 (14.2) | 0.040 |
| DBP (mm Hg) | 62.4 (10.1) | 63.5 (11.0) | 0.300 |
| BMI, body mass index; DBP, diastolic blood pressure; SBP, systolic blood pressure.  Data are presented as means (SDs) or frequencies (%) as appropriate.  †Comparison between groups was conducted using *t* tests or χ^2^ tests. | | | |
|  |  |  |  |
